# 5-Bromo-2-(4-fluoro­phen­yl)-7-methyl-3-phenyl­sulfinyl-1-benzo­furan

**DOI:** 10.1107/S1600536813016760

**Published:** 2013-06-22

**Authors:** Hong Dae Choi, Pil Ja Seo, Uk Lee

**Affiliations:** a Department of Chemistry, Dongeui University, San 24 Kaya-dong, Busanjin-gu, Busan 614-714, Republic of Korea; b Department of Chemistry, Pukyong National University, 599-1 Daeyeon 3-dong, Nam-gu, Busan 608-737, Republic of Korea

## Abstract

In the title compound, C_21_H_14_BrFO_2_S, the dihedral angles between the mean plane [r.m.s. deviation = 0.005 (1) Å] of the benzo­furan ring system and the pendant 4-fluoro­phenyl and phenyl rings are 1.50 (8) and 81.47 (6)°, respectively. In the crystal, mol­ecules are linked by weak C—H⋯O hydrogen bonds into supra­molecular chains running along the *a-*axis direction. A short S⋯O contact [2.9623 (13) Å] involving the sulfinyl groups is observed between inversion-related chains.

## Related literature

For background information and the crystal structures of related compounds, see: Choi *et al.* (2009 ▶); Seo *et al.* (2011 ▶). For details of sulfin­yl–sulfinyl inter­actions, see: Choi *et al.* (2008 ▶). For a review of carbon­yl–carbonyl inter­actions, see: Allen *et al.* (1998 ▶).
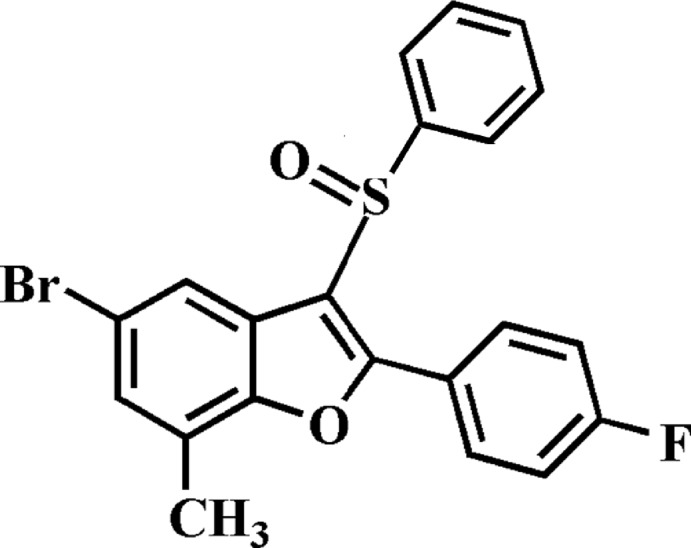


## Experimental

### 

#### Crystal data

C_21_H_14_BrFO_2_S
*M* *_r_* = 429.29
Triclinic, 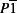

*a* = 7.9961 (2) Å
*b* = 10.6641 (2) Å
*c* = 11.1695 (2) Å
α = 71.980 (1)°
β = 74.694 (1)°
γ = 79.964 (1)°
*V* = 869.13 (3) Å^3^
*Z* = 2
Mo *K*α radiation
μ = 2.51 mm^−1^
*T* = 173 K
0.30 × 0.24 × 0.18 mm

#### Data collection

Bruker SMART APEXII CCD diffractometer
Absorption correction: multi-scan (*SADABS*; Bruker, 2009 ▶) *T*_min_ = 0.523, *T*_max_ = 0.665
20230 measured reflections
4315 independent reflections
3839 reflections with *I* > 2σ(*I*)
*R*_int_ = 0.042

#### Refinement

*R*[*F* ^2^ > 2σ(*F* ^2^)] = 0.029
*wR*(*F* ^2^) = 0.075
*S* = 1.05
4315 reflections
236 parameters
H-atom parameters constrained
Δρ_max_ = 0.41 e Å^−3^
Δρ_min_ = −0.54 e Å^−3^

### 

Data collection: *APEX2* (Bruker, 2009 ▶); cell refinement: *SAINT* (Bruker, 2009 ▶); data reduction: *SAINT*; program(s) used to solve structure: *SHELXS97* (Sheldrick, 2008 ▶); program(s) used to refine structure: *SHELXL97* (Sheldrick, 2008 ▶); molecular graphics: *ORTEP-3 for Windows* (Farrugia, 2012 ▶) and *DIAMOND* (Brandenburg, 1998 ▶); software used to prepare material for publication: *SHELXL97*.

## Supplementary Material

Crystal structure: contains datablock(s) global, I. DOI: 10.1107/S1600536813016760/xu5714sup1.cif

Structure factors: contains datablock(s) I. DOI: 10.1107/S1600536813016760/xu5714Isup2.hkl

Supplementary material file. DOI: 10.1107/S1600536813016760/xu5714Isup3.cml

Additional supplementary materials:  crystallographic information; 3D view; checkCIF report

## Figures and Tables

**Table 1:** Hydrogen-bond geometry (Å, °)

*D*—H⋯*A*	*D*—H	H⋯*A*	*D*⋯*A*	*D*—H⋯*A*
C20—H20⋯O2^i^	0.95	2.33	3.280 (3)	176

Symmetry code: (i) 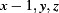.

**Fig. 1.**
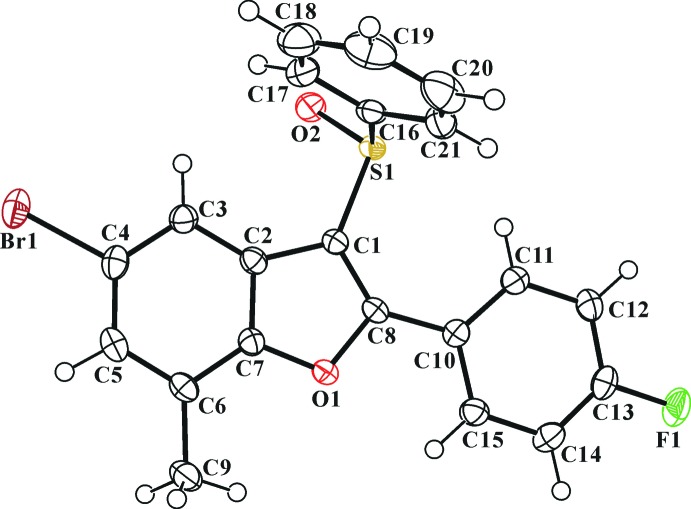
The molecular structure of the title compound with the atom numbering scheme. Displacement ellipsoids are drawn at the 50% probability level. H atoms are presented as small spheres of arbitrary radius.

**Fig. 2.**
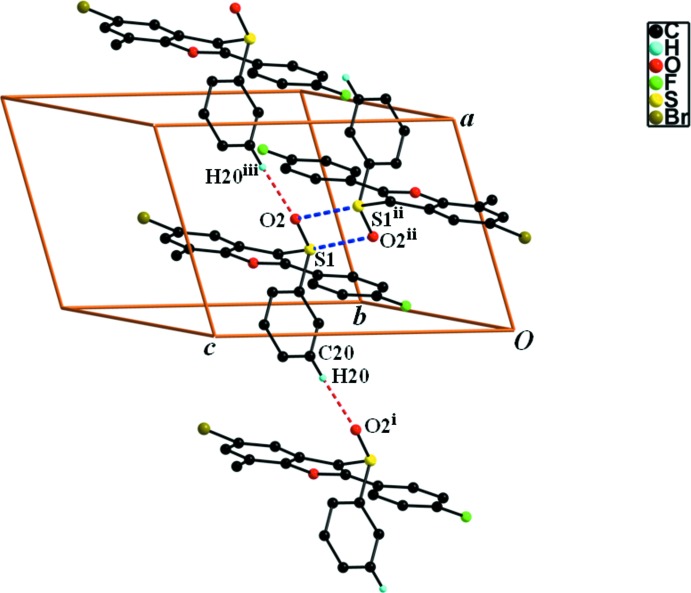
A view of the C—H···O and S···O interactions (dotted lines) in the crystal structure of the title compound. H atoms non-participating in hydrogen-bonding were omitted for clarity. [Symmetry codes: (i) -1+x, y, z; (ii) 1+x, y, z; (iii) - x + 1, - y, - z + 1].

## supplementary crystallographic information

### Comment

As a part of our continuing study of 5-bromo-3-phenylsulfinyl-1-benzofuran derivatives containing phenyl (Choi *et al.*, 2009) and 4-fluorophenyl (Seo *et al.*, 2011) substituents in 2-position, we report herein the crystal structure of the title compound.

In the title molecule (Fig. 1), the benzofuran unit is essentially planar, with a mean deviation of 0.005 (1) Å from the least-squares plane defined by the nine constituent atoms. The dihedral angles between the mean plane of the benzofuran ring system and the pendant 4-fluorophenyl and phenyl rings are 1.50 (8) and 81.47 (6)°, respectively. In the crystal structure (Fig. 2), molecules are connected by weak C—H···O hydrogen bonds (Table 1) into chains extending along the *a-axis* direction, these chains are further packed into stacks by a sulfinyl–sulfinyl interaction (Choi *et al.*, 2008) interpreted as similar to a type-ll carbonyl–carbonyl interaction (Allen *et al.*, 1998), with a S1···O2^ii^ distance of 2.9623 (13) Å [symmetry code: (ii) 1- *x*, - *y*, 1- *z*].

### Experimental

3-Chloroperoxybenzoic acid (77%, 202 mg, 0.9 mmol) was added in small portions to a stirred solution of 5-bromo-2-(4-fluorophenyl)-7-methyl-3-phenylsulfanyl-1-benzofuran (330 mg, 0.8 mmol) in dichloromethane (30 mL) at 273 K. After being stirred at room temperature for 5h, the mixture was washed with saturated sodium bicarbonate solution and the organic layer was separated, dried over magnesium sulfate, filtered and concentrated at reduced pressure. The residue was purified by column chromatography (hexane-ethyl acetate, 2:1 v/v) to afford the title compound as a colorless solid [yield 56%, m.p. 485–486 K; *R*_f_ = 0.72 (hexane–ethyl acetate, 2:1 v/v)]. Single crystals suitable for X-ray diffraction were prepared by slow evaporation of a solution of the title compound in benzene at room temperature.

### Refinement

All H atoms were positioned geometrically and refined using a riding model, with C—H = 0.95 for aryl and 0.98 Å for methyl H atoms. *U*_iso_(H) = 1.2*U*_eq_(C) for aryl and 1.5*U*_eq_(C) for methyl H atoms. The positions of methyl hydrogens were optimized rotationally.

### Crystal data

**Table:**

C_21_H_14_BrFO_2_S	*Z* = 2
*M**_r_* = 429.29	*F*(000) = 432
Triclinic, *P*1	*D*_x_ = 1.640 Mg m^−^^3^
Hall symbol: -P 1	Melting point = 485–486 K
*a* = 7.9961 (2) Å	Mo *K*α radiation, λ = 0.71073 Å
*b* = 10.6641 (2) Å	Cell parameters from 9897 reflections
*c* = 11.1695 (2) Å	θ = 2.4–28.2°
α = 71.980 (1)°	µ = 2.51 mm^−^^1^
β = 74.694 (1)°	*T* = 173 K
γ = 79.964 (1)°	Block, colourless
*V* = 869.13 (3) Å^3^	0.30 × 0.24 × 0.18 mm

### Data collection

**Table:**

Bruker SMART APEXII CCD diffractometer	4315 independent reflections
Radiation source: rotating anode	3839 reflections with *I* > 2σ(*I*)
Graphite multilayer monochromator	*R*_int_ = 0.042
Detector resolution: 10.0 pixels mm^-1^	θ_max_ = 28.4°, θ_min_ = 2.0°
φ and ω scans	*h* = −10→10
Absorption correction: multi-scan (*SADABS*; Bruker, 2009)	*k* = −14→14
*T*_min_ = 0.523, *T*_max_ = 0.665	*l* = −14→14
20230 measured reflections	

### Refinement

**Table:**

Refinement on *F*^2^	Primary atom site location: structure-invariant direct methods
Least-squares matrix: full	Secondary atom site location: difference Fourier map
*R*[*F*^2^ > 2σ(*F*^2^)] = 0.029	Hydrogen site location: difference Fourier map
*wR*(*F*^2^) = 0.075	H-atom parameters constrained
*S* = 1.05	*w* = 1/[σ^2^(*F*_o_^2^) + (0.0333*P*)^2^ + 0.4794*P*] where *P* = (*F*_o_^2^ + 2*F*_c_^2^)/3
4315 reflections	(Δ/σ)_max_ = 0.003
236 parameters	Δρ_max_ = 0.41 e Å^−^^3^
0 restraints	Δρ_min_ = −0.54 e Å^−^^3^

### Special details

**Table:**

Geometry. All esds (except the esd in the dihedral angle between two l.s. planes) are estimated using the full covariance matrix. The cell esds are taken into account individually in the estimation of esds in distances, angles and torsion angles; correlations between esds in cell parameters are only used when they are defined by crystal symmetry. An approximate (isotropic) treatment of cell esds is used for estimating esds involving l.s. planes.
Refinement. Refinement of F^2^ against ALL reflections. The weighted R-factor wR and goodness of fit S are based on F^2^, conventional R-factors R are based on F, with F set to zero for negative F^2^. The threshold expression of F^2^ > 2sigma(F^2^) is used only for calculating R-factors(gt) etc. and is not relevant to the choice of reflections for refinement. R-factors based on F^2^ are statistically about twice as large as those based on F, and R- factors based on ALL data will be even larger.

### Fractional atomic coordinates and isotropic or equivalent isotropic displacement parameters (Å^2^)

**Table:**

	*x*	*y*	*z*	*U*_iso_*/*U*_eq_	
Br1	0.53307 (3)	0.21563 (2)	1.020561 (18)	0.03609 (8)	
S1	0.37815 (5)	0.14799 (4)	0.52980 (4)	0.02275 (10)	
F1	0.03254 (17)	0.61879 (13)	0.03936 (11)	0.0404 (3)	
O1	0.26465 (16)	0.51907 (12)	0.55316 (11)	0.0224 (2)	
O2	0.53338 (17)	0.07965 (13)	0.58227 (14)	0.0314 (3)	
C1	0.3368 (2)	0.30250 (16)	0.56610 (16)	0.0210 (3)	
C2	0.3727 (2)	0.32575 (17)	0.67753 (17)	0.0218 (3)	
C3	0.4376 (2)	0.24909 (18)	0.78483 (17)	0.0245 (3)	
H3	0.4730	0.1570	0.7975	0.029*	
C4	0.4476 (2)	0.31452 (19)	0.87148 (17)	0.0266 (4)	
C5	0.3997 (2)	0.44971 (19)	0.85545 (17)	0.0268 (4)	
H5	0.4103	0.4888	0.9185	0.032*	
C6	0.3367 (2)	0.52804 (18)	0.74877 (17)	0.0236 (3)	
C7	0.3256 (2)	0.46035 (17)	0.66358 (16)	0.0217 (3)	
C8	0.2727 (2)	0.42141 (16)	0.49445 (16)	0.0207 (3)	
C9	0.2845 (3)	0.67413 (19)	0.7268 (2)	0.0319 (4)	
H9A	0.2781	0.7145	0.6363	0.048*	
H9B	0.3711	0.7146	0.7468	0.048*	
H9C	0.1703	0.6887	0.7830	0.048*	
C10	0.2095 (2)	0.46846 (17)	0.37493 (16)	0.0216 (3)	
C11	0.2097 (2)	0.38651 (18)	0.29886 (17)	0.0264 (4)	
H11	0.2505	0.2953	0.3251	0.032*	
C12	0.1508 (3)	0.4366 (2)	0.18517 (18)	0.0287 (4)	
H12	0.1520	0.3810	0.1328	0.034*	
C13	0.0906 (2)	0.56897 (19)	0.15021 (17)	0.0274 (4)	
C14	0.0875 (2)	0.65295 (19)	0.22250 (18)	0.0295 (4)	
H14	0.0446	0.7437	0.1960	0.035*	
C15	0.1482 (2)	0.60225 (18)	0.33472 (17)	0.0258 (4)	
H15	0.1482	0.6592	0.3854	0.031*	
C16	0.1908 (2)	0.07458 (17)	0.64136 (18)	0.0240 (3)	
C17	0.2020 (3)	0.00195 (19)	0.7657 (2)	0.0320 (4)	
H17	0.3086	−0.0096	0.7921	0.038*	
C18	0.0577 (3)	−0.0536 (2)	0.8510 (2)	0.0418 (5)	
H18	0.0643	−0.1030	0.9368	0.050*	
C19	−0.0970 (3)	−0.0370 (2)	0.8114 (3)	0.0456 (6)	
H19	−0.1967	−0.0747	0.8706	0.055*	
C20	−0.1076 (3)	0.0339 (2)	0.6864 (3)	0.0451 (6)	
H20	−0.2138	0.0444	0.6598	0.054*	
C21	0.0378 (3)	0.0897 (2)	0.6001 (2)	0.0323 (4)	
H21	0.0324	0.1376	0.5138	0.039*	

### Atomic displacement parameters (Å^2^)

**Table:**

	*U*^11^	*U*^22^	*U*^33^	*U*^12^	*U*^13^	*U*^23^
Br1	0.03889 (12)	0.04419 (13)	0.02697 (11)	−0.00605 (9)	−0.01581 (8)	−0.00429 (8)
S1	0.0246 (2)	0.0195 (2)	0.0269 (2)	0.00332 (16)	−0.00942 (17)	−0.01036 (16)
F1	0.0500 (7)	0.0448 (7)	0.0276 (6)	−0.0031 (6)	−0.0208 (5)	−0.0026 (5)
O1	0.0284 (6)	0.0190 (6)	0.0217 (6)	−0.0010 (5)	−0.0067 (5)	−0.0080 (5)
O2	0.0268 (6)	0.0291 (7)	0.0435 (8)	0.0083 (5)	−0.0169 (6)	−0.0160 (6)
C1	0.0225 (8)	0.0198 (8)	0.0220 (8)	−0.0013 (6)	−0.0052 (6)	−0.0078 (6)
C2	0.0198 (7)	0.0231 (8)	0.0241 (8)	−0.0029 (6)	−0.0045 (6)	−0.0088 (7)
C3	0.0234 (8)	0.0241 (8)	0.0263 (8)	−0.0025 (7)	−0.0070 (7)	−0.0063 (7)
C4	0.0241 (8)	0.0333 (10)	0.0228 (8)	−0.0071 (7)	−0.0073 (7)	−0.0045 (7)
C5	0.0267 (9)	0.0341 (10)	0.0237 (8)	−0.0087 (7)	−0.0028 (7)	−0.0132 (7)
C6	0.0230 (8)	0.0252 (9)	0.0248 (8)	−0.0069 (7)	−0.0013 (7)	−0.0108 (7)
C7	0.0227 (8)	0.0226 (8)	0.0202 (8)	−0.0042 (6)	−0.0041 (6)	−0.0062 (6)
C8	0.0218 (8)	0.0204 (8)	0.0216 (8)	−0.0022 (6)	−0.0033 (6)	−0.0096 (6)
C9	0.0400 (11)	0.0273 (9)	0.0325 (10)	−0.0052 (8)	−0.0068 (8)	−0.0142 (8)
C10	0.0202 (8)	0.0233 (8)	0.0197 (8)	−0.0020 (6)	−0.0028 (6)	−0.0055 (6)
C11	0.0302 (9)	0.0241 (9)	0.0251 (8)	−0.0003 (7)	−0.0082 (7)	−0.0068 (7)
C12	0.0325 (9)	0.0328 (10)	0.0244 (9)	−0.0046 (8)	−0.0080 (7)	−0.0108 (7)
C13	0.0260 (8)	0.0342 (10)	0.0194 (8)	−0.0041 (7)	−0.0069 (7)	−0.0016 (7)
C14	0.0305 (9)	0.0269 (9)	0.0263 (9)	0.0017 (7)	−0.0068 (7)	−0.0027 (7)
C15	0.0286 (9)	0.0237 (9)	0.0245 (8)	0.0002 (7)	−0.0054 (7)	−0.0080 (7)
C16	0.0263 (8)	0.0166 (8)	0.0320 (9)	0.0005 (6)	−0.0097 (7)	−0.0097 (7)
C17	0.0379 (10)	0.0236 (9)	0.0355 (10)	−0.0036 (8)	−0.0150 (8)	−0.0033 (8)
C18	0.0525 (13)	0.0287 (10)	0.0405 (12)	−0.0096 (10)	−0.0072 (10)	−0.0042 (9)
C19	0.0402 (12)	0.0361 (12)	0.0577 (15)	−0.0130 (10)	0.0039 (11)	−0.0166 (11)
C20	0.0289 (10)	0.0455 (13)	0.0706 (16)	−0.0040 (9)	−0.0139 (11)	−0.0275 (12)
C21	0.0320 (10)	0.0315 (10)	0.0406 (11)	0.0014 (8)	−0.0163 (8)	−0.0157 (8)

### Geometric parameters (Å, º)

**Table:**

Br1—C4	1.9013 (18)	C9—H9C	0.9800
S1—O2	1.4899 (13)	C10—C11	1.394 (2)
S1—C1	1.7723 (17)	C10—C15	1.398 (2)
S1—C16	1.7972 (19)	C11—C12	1.388 (3)
S1—O2^i^	2.9623 (13)	C11—H11	0.9500
F1—C13	1.357 (2)	C12—C13	1.378 (3)
O1—C7	1.373 (2)	C12—H12	0.9500
O1—C8	1.377 (2)	C13—C14	1.372 (3)
C1—C8	1.368 (2)	C14—C15	1.382 (3)
C1—C2	1.448 (2)	C14—H14	0.9500
C2—C7	1.389 (2)	C15—H15	0.9500
C2—C3	1.396 (2)	C16—C17	1.383 (3)
C3—C4	1.381 (3)	C16—C21	1.384 (3)
C3—H3	0.9500	C17—C18	1.378 (3)
C4—C5	1.393 (3)	C17—H17	0.9500
C5—C6	1.388 (3)	C18—C19	1.387 (4)
C5—H5	0.9500	C18—H18	0.9500
C6—C7	1.388 (2)	C19—C20	1.383 (4)
C6—C9	1.501 (3)	C19—H19	0.9500
C8—C10	1.464 (2)	C20—C21	1.389 (3)
C9—H9A	0.9800	C20—H20	0.9500
C9—H9B	0.9800	C21—H21	0.9500
			
O2—S1—C1	105.07 (8)	C11—C10—C15	118.65 (16)
O2—S1—C16	107.03 (8)	C11—C10—C8	123.09 (16)
C1—S1—C16	97.33 (8)	C15—C10—C8	118.26 (15)
O2—S1—O2^i^	79.03 (6)	C12—C11—C10	120.80 (17)
C1—S1—O2^i^	169.13 (7)	C12—C11—H11	119.6
C16—S1—O2^i^	90.93 (6)	C10—C11—H11	119.6
C7—O1—C8	107.06 (13)	C13—C12—C11	118.30 (17)
C8—C1—C2	107.30 (14)	C13—C12—H12	120.8
C8—C1—S1	127.61 (13)	C11—C12—H12	120.8
C2—C1—S1	125.05 (13)	F1—C13—C14	118.48 (17)
C7—C2—C3	119.02 (16)	F1—C13—C12	118.73 (17)
C7—C2—C1	104.82 (15)	C14—C13—C12	122.80 (17)
C3—C2—C1	136.16 (16)	C13—C14—C15	118.36 (17)
C4—C3—C2	116.31 (16)	C13—C14—H14	120.8
C4—C3—H3	121.8	C15—C14—H14	120.8
C2—C3—H3	121.8	C14—C15—C10	121.08 (17)
C3—C4—C5	123.66 (17)	C14—C15—H15	119.5
C3—C4—Br1	118.48 (14)	C10—C15—H15	119.5
C5—C4—Br1	117.85 (13)	C17—C16—C21	121.00 (19)
C6—C5—C4	120.97 (16)	C17—C16—S1	119.87 (14)
C6—C5—H5	119.5	C21—C16—S1	119.11 (15)
C4—C5—H5	119.5	C18—C17—C16	119.7 (2)
C7—C6—C5	114.56 (16)	C18—C17—H17	120.2
C7—C6—C9	122.50 (17)	C16—C17—H17	120.2
C5—C6—C9	122.94 (16)	C17—C18—C19	119.8 (2)
O1—C7—C6	123.77 (15)	C17—C18—H18	120.1
O1—C7—C2	110.76 (14)	C19—C18—H18	120.1
C6—C7—C2	125.47 (16)	C20—C19—C18	120.6 (2)
C1—C8—O1	110.06 (15)	C20—C19—H19	119.7
C1—C8—C10	136.22 (16)	C18—C19—H19	119.7
O1—C8—C10	113.71 (14)	C19—C20—C21	119.7 (2)
C6—C9—H9A	109.5	C19—C20—H20	120.1
C6—C9—H9B	109.5	C21—C20—H20	120.1
H9A—C9—H9B	109.5	C16—C21—C20	119.2 (2)
C6—C9—H9C	109.5	C16—C21—H21	120.4
H9A—C9—H9C	109.5	C20—C21—H21	120.4
H9B—C9—H9C	109.5		
			
O2—S1—C1—C8	146.26 (16)	S1—C1—C8—C10	3.3 (3)
C16—S1—C1—C8	−103.83 (17)	C7—O1—C8—C1	0.28 (18)
O2^i^—S1—C1—C8	35.4 (4)	C7—O1—C8—C10	179.29 (14)
O2—S1—C1—C2	−31.09 (17)	C1—C8—C10—C11	−2.7 (3)
C16—S1—C1—C2	78.82 (16)	O1—C8—C10—C11	178.64 (15)
O2^i^—S1—C1—C2	−142.0 (3)	C1—C8—C10—C15	178.07 (19)
C8—C1—C2—C7	0.15 (19)	O1—C8—C10—C15	−0.6 (2)
S1—C1—C2—C7	177.95 (13)	C15—C10—C11—C12	0.3 (3)
C8—C1—C2—C3	−180.0 (2)	C8—C10—C11—C12	−178.96 (17)
S1—C1—C2—C3	−2.2 (3)	C10—C11—C12—C13	−0.7 (3)
C7—C2—C3—C4	0.9 (2)	C11—C12—C13—F1	−179.89 (17)
C1—C2—C3—C4	−178.93 (19)	C11—C12—C13—C14	0.4 (3)
C2—C3—C4—C5	−1.0 (3)	F1—C13—C14—C15	−179.41 (16)
C2—C3—C4—Br1	179.48 (13)	C12—C13—C14—C15	0.3 (3)
C3—C4—C5—C6	0.3 (3)	C13—C14—C15—C10	−0.8 (3)
Br1—C4—C5—C6	179.83 (14)	C11—C10—C15—C14	0.5 (3)
C4—C5—C6—C7	0.4 (3)	C8—C10—C15—C14	179.73 (16)
C4—C5—C6—C9	−179.60 (17)	O2—S1—C16—C17	17.79 (17)
C8—O1—C7—C6	−179.86 (16)	C1—S1—C16—C17	−90.49 (15)
C8—O1—C7—C2	−0.18 (19)	O2^i^—S1—C16—C17	96.59 (15)
C5—C6—C7—O1	179.15 (15)	O2—S1—C16—C21	−160.92 (14)
C9—C6—C7—O1	−0.8 (3)	C1—S1—C16—C21	90.81 (15)
C5—C6—C7—C2	−0.5 (3)	O2^i^—S1—C16—C21	−82.11 (14)
C9—C6—C7—C2	179.53 (17)	C21—C16—C17—C18	−1.7 (3)
C3—C2—C7—O1	−179.89 (15)	S1—C16—C17—C18	179.67 (15)
C1—C2—C7—O1	0.02 (19)	C16—C17—C18—C19	0.5 (3)
C3—C2—C7—C6	−0.2 (3)	C17—C18—C19—C20	0.5 (3)
C1—C2—C7—C6	179.69 (17)	C18—C19—C20—C21	−0.4 (3)
C2—C1—C8—O1	−0.27 (19)	C17—C16—C21—C20	1.8 (3)
S1—C1—C8—O1	−178.00 (12)	S1—C16—C21—C20	−179.50 (15)
C2—C1—C8—C10	−178.96 (19)	C19—C20—C21—C16	−0.8 (3)

Symmetry code: (i) −*x*+1, −*y*, −*z*+1.

### Hydrogen-bond geometry (Å, º)

**Table:**

*D*—H···*A*	*D*—H	H···*A*	*D*···*A*	*D*—H···*A*
C20—H20···O2^ii^	0.95	2.33	3.280 (3)	176

Symmetry code: (ii) *x*−1, *y*, *z*.
